# Recent miRNA Research in Asthma

**DOI:** 10.1007/s11882-022-01050-1

**Published:** 2022-12-02

**Authors:** Rinku Sharma, Anshul Tiwari, Michael J. McGeachie

**Affiliations:** Channing Division of Network Medicine, Brigham and Women’s Hospital, Harvard Medical School, Boston, MA USA

**Keywords:** miRNA, Asthma, Review

## Abstract

**Purpose of Review:**

The study of microRNA in asthma has revealed a vibrant new level of gene regulation underlying asthma pathology. Several miRNAs have been shown to be important in asthma, influencing various biological mechanisms which lead to asthma pathology and symptoms. In addition, miRNAs have been proposed as biomarkers of asthma affection status, asthma severity, and asthma treatment response. We review all recent asthma-miRNA work, while also presenting comprehensive tables of all miRNA results related to asthma.

**Recent Findings:**

We here reviewed 63 recent studies published reporting asthma and miRNA research, and an additional 14 reviews of the same. We summarized the information for both adult and childhood asthma, as well as research on miRNAs in asthma–COPD overlap syndrome (ACOs), and virus-induced asthma exacerbations.

**Summary:**

We attempted to present a comprehensive collection of recently published asthma-associated miRNAs as well as tables of all published asthma-related miRNA results.

## Introduction

Asthma is one of the most prevalent non-communicable diseases, and it has a significant influence on many people’s quality of life. It affects 23.4 million people in the USA alone (5–10% of the population), including 7 million children [1]. It affects an estimated 300 million people worldwide, with another 100 million predicted to be impacted by 2025 [2]. The World Health Organization (WHO) estimates that 15 million disability-adjusted life-years are lost each year, with 250,000 asthma fatalities reported globally [3]. Asthma is the 16th greatest cause of years lived with disability and the 28th leading source of disease burden, as defined by disability-adjusted life years, globally.

Asthma is a multifaceted condition with a complex etiology involving interactions between genetic susceptibility, host factors, and environmental exposures. Environmental factors may involve exposure to air pollution, pollens, mold, aeroallergens, tobacco smoke, etc., while host factors include obesity, nutrition, infections, allergic sensitization, etc. Genetic factors include asthma susceptibility loci on genes or family history of asthma. Although the precise mechanisms of asthma are unknown, they may include airway inflammation, airway tone control, and airway responsiveness [4]. Asthma has been studied at multiple omics levels, encompassing genomes, metabolomics, epigenomics, and transcriptomics, and therefore is associated with complex cellular and genomic interactions [5, 6]. RNAs have traditionally been regarded to be molecules that solely encode genetic information for protein production, while gene modulation and cell-signaling networks have been thought to be the key regulatory systems in cells. However, following relatively recent breakthroughs in non-coding RNAs, such as the discovery of microRNAs (miRNAs), this paradigm is evolving. miRNAs are 18–22 nucleotides long and stop protein translation by interacting with mRNA [7]. Each miRNA may affect hundreds to thousands of genes and, when taken in aggregate, may lead to a combinatorial increase in regulatory complexity. Even though miRNAs were discovered about 30 years ago, their immense role in the immune system has only begun to be appreciated. miRNAs’ role has been deeply studied in several human diseases including cancer, skin conditions, and several lung disorders, including but not limited to idiopathic pulmonary fibrosis (IPF), cystic fibrosis (CF), chronic obstructive pulmonary disease (COPD), asthma, and pulmonary illness [8, 9]. In asthma, miRNAs regulate multiple pro-inflammatory pathways as well as smooth muscle cell proliferation, driving airway hyperresponsiveness and contributing to the disease’s development [10–13].

In the year leading up to this review, 63 research articles on asthma and miRNAs were published, with another 14 reviews. Here, we contribute to the previously remarkable collection of work by summarizing the quite remarkable body of work on the impact of miRNAs in asthma. Our purpose is to review the recent contributions to the field of miRNA in asthma; prior work has already been reviewed, and we refer the interested reader to other excellent reviews [10, 13–18, 19••, 20, 21, 22••, 23–25]. However, we have attempted to provide comprehensive treatment of the entire body of literature in our figures and tables, which we hope will be a useful reference for miRNA researchers doing work in asthma.

### Asthma and miRNA

Asthma has a high degree of variability among patients, making it difficult to develop diagnostic and therapeutic tools. Chronic airway inflammation, mucus hyper-secretion, and bronchial hyper-responsiveness, as well as respiratory symptoms such as wheezing, shortness of breath, chest tightness, and cough, are all hallmarks of asthma. Asthma can further be classified into distinct mechanistic pathways or endotypes based on variable clinical presentations or phenotypes [26]. Using induced sputum or peripheral blood cytology to phenotype and endotype asthma can help with treatment responsiveness, identifying pathogenic pathways, and anticipating complications. Moreover, asthma shifts significantly throughout the lifespan. Childhood asthma is characterized by having a high general frequency, a male predominance prior to puberty, frequent remission, and rare fatality. Female preponderance, exceptional remission, and atypical mortality are all characteristics of adult asthma [27]. The longevity of asthma symptoms, medication use, lung function, low socioeconomic status, racial/ethnic minorities, and a neutrophilic phenotype have all been linked to the severity of childhood asthma. Increased IgE, elevated FeNO, eosinophilia, obesity, smoking, and low socioeconomic status have all been linked to adult asthma severity [28]. Despite higher prebronchodilator FEV1/FVC, adult-onset illness is related to more respiratory symptoms and asthma medication use [28]. Adult-onset asthma is less quiescent and appears to be more stable than childhood-onset asthma, with more relapses and fewer remissions. These characteristics reflect the complexity of asthma and the various elements involved in its pathophysiology.

A layer of regulation by miRNA adds to the regulatory network governing genetics, epigenetics, protein synthesis, and immune response in asthma. miRNAs are short non-coding RNAs that regulate gene expression by binding to target messenger RNAs and causing mRNA degradation or translational repression [29]. miRNAs can also regulate epigenetic DNA modifications, while also being influenced by epigenetic modifications [20, 30]. miRNAs play broadly different roles based on their location in the organism: (1) extracellular miRNAs are found inside extracellular vesicles such as exosomes, macrovesicles, and apoptotic bodies, which may act as cell-to-cell or system-to-system messengers, and (2) intracellular miRNAs, which govern protein production internal to a cell [31]. Intracellular miRNAs govern a variety of cellular pathways, and because their expression varies by tissue and disease, they have been widely exploited as prognostic and diagnostic biomarkers for a variety of disorders, including viral infections, cancer, cardiovascular disease, and allergic diseases [32, 33]. Extracellular, or circulating miRNAs, have also been investigated as potential biomarkers as they are resistant to degradation and ubiquitination [34].

### Childhood Asthma and miRNA

**Fig. 1:**
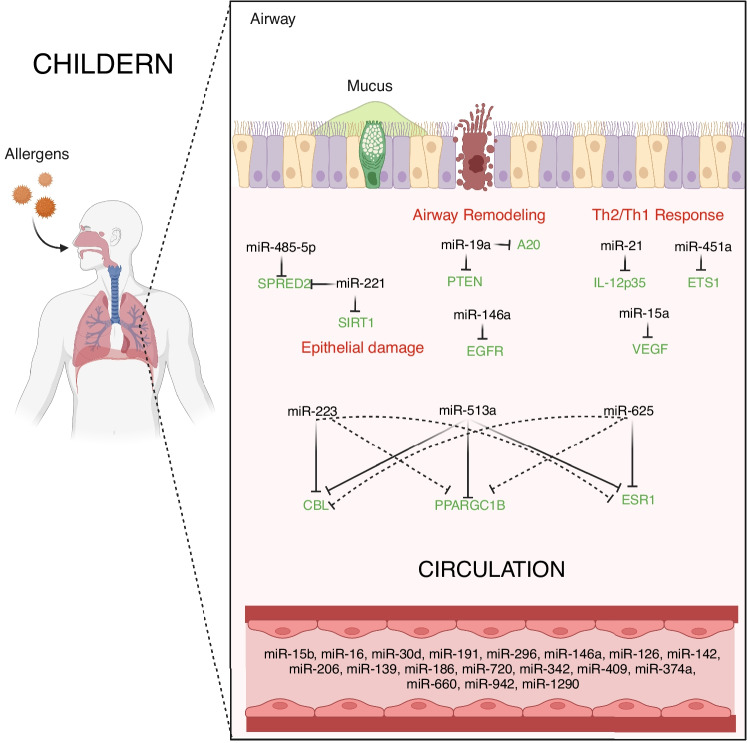
Illustrating miRNAs and their target genes associated with childhood asthma

Allergic asthma may start as early as childhood, with up to 50% of adults reporting symptoms as children [35]. The composition of miRNAs in circulation and their potential as asthma biomarkers have been studied [17, 22••]. For instance, changes in miR-196a-2 expression and serum ANXA1 levels may play a role in asthma etiology. Furthermore, ANXA1 and miR-196a-2 could be used as diagnostic biomarkers for asthma and therapeutic targets in the future [36]. Wang et al. showed that deregulated miR-451a-ETS1 axis is a unique molecular mechanism responsible for pediatric asthma pathogenesis [37]. A study with CAMP data showed baseline FEV1/FVC and miR-221-5p were independent predictors of asthma remission by early adulthood [38]. Another study revealed reduced expression of miR-145-5p as a risk factor for early decline of long-term lung function growth leading to adult COPD in children with asthma and additionally increases airway smooth muscle cell proliferation [39]. A study showed that the aberrant expression of immune-related miRNAs (miR-146a and miR-106b) and inflammatory cytokines (IL-5 and IL-13) among asthmatic children led to their probable role in asthma pathogenesis [40]. Cancer-related long non-coding RNAs (lncRNA) were negatively correlated with miR-33a and miR-495 and positively with inflammatory cytokines in asthmatic children [41]. Another study on lncRNA showed that a lncRNA, RMRP, plays a pro-inflammatory and pro-fibrotic effect in pediatric asthma through targeting the miR-206/CCL2 axis [42]. Tiwari et al. investigated the association of circulating miRNAs from asthmatic children with seasonal variation in allergic inflammation and asthma symptoms and found that miR-328-3p and let-7d-3p expression varies seasonally and are significantly associated with seasonal asthma symptoms and seasonal allergies where let-7d-3p plays a potentially protective role and miR-328-3p has a deleterious role in asthmatic children sensitized to mulberry [43]. miR-15a is expressed during human lung development, is influenced by intrauterine smoke exposure, regulates the intrauterine expression of asthma genes, and is associated to asthma severity [44]. A study showed that baicalin regulates the onset of asthma in children by up-regulating miR-103 and modulating the TLR4/NF-B pathway [45]. After demonstrating that many miRNAs are altered in asthma, more research is needed to mechanistically characterize their role(s) in childhood asthma etiology (Table 1, Fig. 1).

**Table 1:** List of childhood asthma associated miRNAs

**Adult/childhood**	**miRNA ID**	**Target gene**	**Function**	**Sample**	**Reference (PubMed ID)**	**Review/research**
Childhood	miR-145-5p	NA	Associated with the early decline patterns of lung function growth leading to COPD in children with asthma and additionally increases airway smooth muscle cell proliferation	Serum	33385444	Research
Childhood	miR-196a2	ANXA1	miR-196a2 expression and serum ANXA1 concentration may play a role in the pathogenesis of asthma	Serum	32279913	Research
Childhood	miR-15a	NA	Role in the fetal origin of asthma	Fetal lung	33291534	Research
Childhood	miR-146a, miR-106b	NA	Aberrant expression of immune-related microRNAs in pediatric patients with asthma	Plasma	33688482	Research
Childhood	miR-33a, miR-495	NA	lncRNAs correlated negatively with miR-33a and miR-495 and positively with inflammatory cytokines in asthmatic children	Blood	34288494	Research
Childhood	miR-328-3p, let-7d-3p	NA	Seasonal variation in miR-328-3p and let-7d-3p are associated with seasonal allergies and asthma symptoms in children	Serum	34212545	Research
Childhood	miR-103	NA	Baicalin regulates the onset of asthma in children by up-regulating microRNA-103 and modulating the TLR4/NF-B pathway	Mouse	33730981	Research
Childhood	miR-206	CCL2	Pro-inflammatory and pro-fibrotic role of lncRNA RMRP in pediatric asthma through targeting microRNA-206/CCL2 axis	Pulmonary tissue	33511814	Research
Childhood	miRs:221-5p, 139-3p, 96-5p, 6641-5p, 199b-5p, 151b, 1307-3p, 148a-5p	NA	Childhood asthma remission	Serum	32888944	Research
Childhood	miR-451a	ETS1	Down-regulation of miRNA-451a promotes the differentiation of CD4^+^ T cells toward Th2 cells by up-regulating ETS1 in childhood asthma	Lymphocytes	33271553	Research
Childhood	miR-192	CXCR5	Decreased miR-192 in blood of asthmatics	NA	32777705	Review
Childhood	miR-27b-3p	SYK, EGFR, IL-12	Modulation of PI3K-Akt signaling pathway	Blood	33460581	Review
Childhood	miR-143a	NA	Regulation of polymorphonuclear neutrophil counts	Sputum	33460581	Review
Childhood	miR-223a	NA	Attenuation of the airway neutrophil responses	Sputum	33460581	Review
Childhood	miR-21	IL-12p35	Production and activation of inflammatory cells	Serum	33460581	Review
Childhood	miR-221	Spred	Regulation of mast cells functions	Blood	33460581	Review
Childhood	miR-485-3p	NA	Regulation of airway hyperresponsiveness	Blood	33460581	Review
Childhood	miR-21-5p	IL-12	Dysregulation of Th1/Th2 production	Bronchial epithelial cells	33460581	Review
Childhood	miR-146a-3p	NA	Up-regulation of small airway reversibility	Bronchial epithelial cells	33460581	Review
Childhood	miR-155-5p	CCL11, CCL26, IL-13	Inhibition of eosinophil production	Bronchial epithelial cells	33460581	Review
Childhood	miR-485-3p	SPRED-2	Airway remodeling by decreasing sprout-related EVH1 domain-containing protein (spred)-2 expression to promote growth factor-mediated Ras/ERK activation	ASMCs	34359876	Review
Childhood	miR-155	NA	A biomarker of worsened lung function	Serum/plasma	33478047	Review
Childhood	miR-16	NA	A significant negative correlation with FEV1	Serum/plasma	33478047	Review
Childhood	miR-199a-5p	NA	Increased in plasma and sputum of patients with neutrophilic asthma. Negative correlation with pulmonary function	Serum/plasma	33478047	Review
Childhood	miR-146b, miR-206, miR-720	NA	NF-kβ and GSK3/AKT pathways, might improve the accuracy of asthma exacerbation risk prediction in a pediatric asthma	Serum	33214212	Review
Childhood	miR-15b, miR-126, miR-139, miR-142, miR-186, miR-191, miR-342, miR-374a, miR-409, miR-660, miR-942, miR-1290	NA	Correlating to lung function parameters in children	Blood	33128813	Review
Childhood	miR-16, miR-30d, miR-296	NA	Correlating to bronchial hyper-responsiveness	Blood	33128813	Review
Childhood	miR-146a, miR-206, miR-720	NA	Potential asthma prediction markers	Blood	33128813	Review
Childhood	miR-223, miR-513a, miR-625	CBL, PPARGC1B, ESR1	Dust mite allergic asthma associated	Blood	33128813	Review
Childhood	miR-15a	VEGF	Low levels in CD4^+^ T cells in pediatric asthma	NA	33128813	Review
Childhood	miR-21	IL-12p35	Predicts therapeutic response to ICS in asthma	NA	32777705	Review
Childhood	miR-146a	EGFR	Up-regulation of miR‑146a inhibits proliferation and promotes apoptosis of ASMCs in asthma	NA	32777705	Review
Childhood	miR-221	SIRT1	Overexpression of miR-221 by targeting SIRT1 induces apoptosis and inhibits proliferation in bronchial epithelial BEAS2B cells	NA	32777705	Review
Childhood	miR-19a	PTEN, A20	Increased in airway T cells Reduction in smooth muscle cells leads to enhanced remodeling	ASMCs	33128813	Review
Childhood	miR-485-5p	SPRED2	Pediatric asthma	NA	33488613	Review
Childhood	miR-221	SPRED, SIRT1	Pediatric asthma	NA	33488613	Review

### Adult Asthma and miRNA

Numerous miRNAs have also been detected in adult asthma studies, which may help in better understanding the disease. One of the studies on RNA samples from eosinophils of individuals with atopic dermatitis, atopy, allergic rhinitis, and asthma identified 18 miRNAs (miR-1276, miR-29B2, miR-3175, miR-33B, miR-4308, miR-4523, miR-4673, miR-4785, miR-590, miR-638, miR-614, miR-142, miR-3064, miR-4434, miR-1304, miR-2355, miR-26A2, and miR-645) differentially expressed in eosinophil samples in cases of atopic dermatitis or asthma, or according to PC20 or IgE levels, compared to healthy samples [11]. According to a meta-analysis, the miR-499 rs3746444 (T > C) polymorphism is associated to asthma susceptibility, while the miR-146a rs2910164 (G > C) polymorphism is protective against asthma susceptibility [46]. A study found that c-kit + cells reduce asthma-related pathologies, likely through modulating miR-126 and miR-133 production [47]. miR-139 can decrease the inflammatory response of Th2 cells by down-regulating the Notch pathway and encouraging bone marrow-derived mesenchymal stem cells into asthmatic lung tissues [48] (Table 2).

**Table 2:** List of adult asthma-associated miRNAs

**Adult/childhood**	**miRNA**	**Target gene**	**Function**	**Sample**	**Reference**	**Review/research**
Adult	miR-21	NA	Promotes oxidative stress and inflammatory responses in asthmatic mice via the DDAH1/Wnt/β-catenin signaling axis	ASMCs	34377230	Research
Adult	miR-23b	Smad3	Controlling TGF-β1-induced airway smooth muscle cell proliferation by regulating Smad3 and, thereby reducing airway remodeling	ASMCs	33152094	Review
Adult	miR-140-3p	IL-13	Down-regulation of miR-140-3p is a cause of the interlukin-13-induced up-regulation of RhoA protein in bronchial smooth muscle cells	ASMCs	33427568, 33385215	Research
Adult	miR-143-3p	TGF-β1, CDK4, Cyclin D1	Overexpression of miR-143-3p could decrease asthma airway remodeling by suppressing proliferation and ECM protein deposition in TGF-β1-mediated airway smooth muscle cells via the negative regulation of NFATc1 signaling	ASMCs	33454598	Review
Adult	miR-223	IGF-1R, TGF-β1	Overexpression of miR-223 could decrease the expression of proteins involved in the extracellular matrix, such as α-SMA (ACTA2), and type I and III collagens	ASMCs	33454598	Review
Adult	miR-21	TGF-beta, Smad7	miR-21-transforming growth factor β1-Smad7 axis modulates the pathogenesis of ovalbumin-induced chronic asthma in human bronchial smooth muscle cells	ASMCs	33601867	Research
Adult	miR-149	TRPM7	miR-149 attenuates the proliferation and migration of TGF-β1-induced airway smooth muscle cells by targeting TRPM7 and affecting downstream MAPK signal pathway	ASMCs	33284571	Research
Adult	miR-140-3p	CD38	Down-regulation of miR-140-3p contributes to up-regulation of CD38 protein in bronchial smooth muscle cells	ASMCs	33121100	Research
Adult	miR-204-5p	TGF-β1	miR-204-5p could reduce ECM production of airway smooth muscle cells by regulating Six1 in asthma	ASMCs	33454598	Review
Adult/Childhood	miR-19a	PTEN, A20	Increased in airway T cells Reduction in smooth muscle cells leads to enhanced remodeling	ASMCs	33128813	Review
Adult	miR-370	FGF1	Exosomes generated from M2 macrophages carry miR-370, which slows asthma progression by inhibiting FGF1 production and the MAPK/STAT1 signaling pathway	Bone marrow cells/exosomes	33994863	Research
Adult	miR-126 and miR-133	NA	c-kit + cells could reduce asthma-related pathologies, likely through modulating miRNA-126 and -133 production	Bone marrow–derived c-kit + cells/serum	33995948	Research
Adult	miR-141-3p	NA	Epithelial miR-141 regulates IL-13-induced airway mucus production	Bronchial epithelial brushing/HBECs and mouse lung tissue	33682796	Research
Adult	miR-206	CD39	Epithelial miR-206 targets CD39/extracellular ATP to up-regulate airway IL-25 and TSLP in type 2-high asthma	Bronchial epithelial brushings	33945508	Research
Adult	miR-30a-3p, miR-30d-3p	NA	Potential role for epithelial circRNA-miRNA-mRNA network in the pathogenesis of asthma	Bronchial epithelial brushings	34336929	Research
Adult	miR-146a	IRAK1	Down-regulated in bronchial brushing samples of asthma patients, inhibits IL-8 and CXCL1 expression and neutrophil migration	Bronchial epithelial brushings	33128813	Review
Adult	miR-629-3p, miR-223-3p, miR-142-3p	NA	Neutrophilic inflammation	Bronchoalveolar lavage fluid	33478047	Review
Adult	miR-185	NA	A potential predictor of asthma severity in blood sera	Circulating eosinophils	33128813	Review
Adult	18 pri-miRs. miRs: 1276, 29B2, 3175, 33B, 4308, 4523, 4673, 4785, 590, 638, 614, 142, 3064, 4434, 1304, 2355, 26A2, 645	NA	Differentially expressed in eosinophil samples in cases of atopic dermatitis or asthmatic condition, or according to PC20 or IgE levels, compared to healthy person samples	Eosinophils	33260893	Research
Adult	miR-221	NA	Decreased levels in epithelial and sputum was associated with eosinophilic airway inflammation in asthma	Epithelial and sputum	33128813	Review
Adult	miR-122-5p	NA	Plasma miR-122-5p can sub-differentiate different types of asthma, such as neutrophilic versus eosinophilic asthma, given its IPA-predicted role in lymphocyte differentiation and function	Extracellular vesicles from plasma	32627209	Research
Adult	miR-200b-3p	SOCS1	A-to-I editing of miR-200b-3p in airway cells is associated with moderate-to-severe asthma	Human bronchial epithelial cells (HBECs)	33446603	Research
Adult	let-7i, miR-423	NA	PRMT1 as a coactivator for STAT1 or RUNX1, which is essential for the transcription of pri-let-7i and pri-miR-423 in epithelial cells and might be relevant to epithelium dysfunction in asthma	Human bronchial epithelial cells (HBECs)	33239422	Research
Adult	miR-143-3p	NA	LncRNA OIP5‑AS1 aggravates house dust mite‑induced inflammatory responses in human bronchial epithelial cells via the miR‑143‑3p/HMGB1 axis	Human bronchial epithelial cells (HBECs)	33174035	Research
Adult	miR-181b-5p	SPP1	miR-181b-5p has been identified as a potential biomarker for airway eosinophilia, and controls pro-inflammatory cytokine release by targeting the secreted phosphoprotein 1 (SPP1) gene	Human bronchial epithelial cells (HBECs)/plasma	33152094	Review
Adult	miR-146a-5p	TRAF6	miR-146a-5p inhibits the inflammatory response and injury of airway epithelial cells via targeting TNF receptor-associated factor 6	Human small airway epithelial cells (HSAECs)	34002665	Research
Adult	miR-149	NA	LncRNA PVT1 exacerbates the inflammation and cell-barrier injury during asthma by regulating miR-149	Human small airway epithelial cells (HSAECs)	32830409	Research
Adult	miR-221-3p	NA	miR-221-3p correlates with eosinophils	Induced sputum	33478047	Review
Adult	miR-98	NA	miR-98 reduces nerve growth factor expression in nicotine-induced airway remodeling	Lung fibroblasts	3308240	Research
Adult	miR-20a-5p	ATG7	miR-20a-5p targets ATG7-regulated cell death, fibrosis, and inflammation	Lung tissue	33684878	Research
Adult	miR-135a	NA	miR-135a inhibits airway inflammatory response in asthmatic mice via regulating JAK/STAT signaling pathway	Lung tissue	33470387	Research
Adult	miR-139	NA	miR-139 can down-regulate the Notch pathway and promote bone marrow-derived mesenchymal stem cells homing in asthmatic lung tissues, thus suppressing the inflammatory response of Th2 cells through immune regulation	Lung tissue	33504414	Research
Adult	let-7a	IL-13	Abundant in the lungs and regulates IL-13 expression	Lung tissue	33128813	Review
Adult	miR-155	NA	Down-regulated in the lymphocytes of allergic asthmatics during pollen season	Lymphocytes	33128813	Review
Adult	miR-210	NA	Increases in human mast cells following IgE sensitization	Mast cells	33128813	Review
Adult	miR-146a, miR-499	NA	Association of two polymorphisms of miRNA-146a rs2910164 (G > C) and miRNA-499 rs3746444 (T > C) with asthma: a meta-analysis	Meta-analysis	32308092	Research
Adult	miR-181b	HMGB1	LncRNA TUG1 promotes airway remodeling and mucus production in asthmatic mice through the microRNA-181b/HMGB1 axis	Mouse model	33640857	Research
Adult	miR‑106b‑5p	SIX1	miR‑106b‑5p targeting SIX1 inhibits TGF‑β1‑induced pulmonary fibrosis and epithelial‑mesenchymal transition in asthma through regulation of E2F1	Mouse model	33495833	Research
Adult	miR-26a, miR-142-3p	NA	Borneol reduces asthma symptoms by inhibiting CD4^+^ T-cell proliferation by down-regulating miR-26a and miR-142-3p	Mouse model	33272847	Research
Adult	miR-15a-5p/miR-29c-3p	NA	Exposure to ozone impacted Th1/Th2 imbalance of CD (4 +) T cells and apoptosis of ASMCs underlying asthmatic progression by activating lncRNA PVT1-miR-15a-5p/miR-29c-3p signaling	Mouse model	33223504	Research
Adult	miR-21	NA	miR-21 inhibition suppresses alveolar M2 macrophages in an ovalbumin-induced allergic asthma mice model	Mouse model	33474864	Research
Adult	miR-21-5p	Smad7	MiR-21-5p in macrophage-derived exosomes targets Smad7 to promote epithelial mesenchymal transition of airway epithelial cells	Mouse model	34040396	Research
Adult	miR-155	NA	TDI (toluene 2,4-diisocyanate)-induced airway inflammation and hyperresponsiveness in asthma	Mouse model/human cell lines	32499335	Research
Adult	miR-21	NA	Dysregulated in circulation and lungs in allergic experimental murine models and human allergic asthmatics	Murine model	33128813	Review
Adult	let-7a, miR-21, miR-133a, miR-155, miR-328, miR-1248	NA	Decreased in exhaled breath condensates from asthmatic compared to healthy subjects	NA	33128813	Review
Adult	miR-16	ADRB2	Negatively correlates to lung function parameters	NA	33128813	Review
Adult	miR-1248	IL-5	Interacts with the 3′UTR to promote IL-5 expression	NA	33128813	Review
Adult	miR-150	eIF4E/Akt	Malat1 up-regulated in airway smooth muscle cells stimulated with platelet-derived growth factor BB (PDGF-BB). Silencing of Malat1 using miR-150 and block of eIF4E/Akt signaling inhibits PDGF-BB-induced airway smooth muscle cells proliferation and migration	NA	32777705	Review
Adult	miR125a	NA	Expression of ANRIL/miR-125a used to investigate the disease exacerbation, exacerbation severity, and inflammation for asthma has a discriminant value	NA	32777705	Review
Adult	miR-1248	IL-5	Elevates Th2 cytokine levels	NA	32777705	Review
Adult	miR-371, miR-138, miR-544, miR-145, miR-214	Runx3	miRNAs capable of combinatorial regulation of Runx3, modulates Th1/Th2 balance in asthma	NA	32777705	Review
Adult	miR-98	TSP1, IL-13	miR-98 suppresses TSP1 expression in peripheral B cells of allergic asthmatics	NA	32777705	Review
Adult	miR19a	TGFbR2	miR-19a targets TGFbR2 gene in severe asthma enhances proliferation of bronchial epithelial cells	NA	32777705	Review
Adult	miR-21, miR-126	IL-13	miRNAs increased in asthmatics compared to controls, expression in bronchial epithelia of asthmatics positively correlated with IL-13	NA	32777705	Review
Adult	miR-221	NA	Asthmatics and OVA-induced allergic mice have miR-221 up-regulated, reduced airway inflammation	NA	32777705	Review
Adult	miR-1165-3p	NA	Circulating miR-1165-3p useful as a biomarker of asthma	NA	32777705	Review
Adult	miR-221-3p	CXCL17	miR-221-3p up-regulates anti-inflammatory chemokine CXCL17, protective against airway eosinophilic inflammation	NA	32777705	Review
Adult	miR-142-3p	NA	miR-142-3p regulates the balance between proliferation and differentiation of ASMCs	NA	32777705	Review
Adult	miR-26a, Let-7a, Let-7d, mir-323, miR-21	NA	Biomarkers for diagnosis of asthma	NA	32777705	Review
Adult	miR-17	NA	Biomarker for the diagnosis of asthma	NA	32777705	Review
Adult	let-7a	IL-13	let-7a useful as a biomarker to discriminate between asthma phenotypes. exogenous let-7 mimic by targeting IL-13 alleviates asthmatic phenotype in OVA allergic mice	NA	32777705	Review
Adult	miR-200	NA	Asthma biomarker	NA	33488613	Review
Adult	miR-346	IL13	Airway inflammation, T helper cell differentiation	NA	33488613	Review
Adult	miR-574-5p	IL5RA	NA	NA	33488613	Review
Adult	miR-24	IL-4 production pathway	Cytokine regulation	NA	33488613	Review
Adult	miR-27	GATA3	NA	NA	33488613	Review
Adult	miR-16	NA	Asthma biomarker	NA	33488613	Review
Adult	miR-125b	NA	NA	NA	33488613	Review
Adult	miR-133b	NA	NA	NA	33488613	Review
Adult	miR-206	NA	NA	NA	33488613	Review
Adult	miR-144-5p	NA	Asthma biomarker	NA	33488613	Review
Adult	let-7 family	NA	Asthma biomarker	NA	33488613	Review
Adult	miR-185-5p	NA	NA	NA	33488613	Review
Adult	miR-320a	NA	NA	NA	33488613	Review
Adult	miR-1246	NA	NA	NA	33488613	Review
Adult	miR-21	IL12p3, IRF5, CSF1R	Imbalance Th1/Th2 response, macrophage M2 polarization	NA	33488613	Review
Adult	miR-142-3p	MAPK, NOD-like receptor, Toll-like receptor, JAKSTAT, and the TGF-b signaling pathways	Neutrophilic asthma	NA	33488613	Review
Adult	miR-223-3p	NA	NA	NA	33488613	Review
Adult	miR-629-3p	NA	NA	NA	33488613	Review
Adult	miR-221-3p	CXCL17	Regulation of eosinophil counts and ROS production	NA	33488613	Review
Adult/Childhood	miR-196a2	NA	miR-196a2 polymorphisms have also been shown to be involved in controlling asthma	NA	33152094	Review
Adult/Childhood	miR-21, miR-223, miR-146a, miR-146b, miR-15	NA	Asthma-related diseases such as atopic dermatitis and allergic rhinitis suggesting a key role in the atopic march from childhood to adulthood	NA	34134446	Review
Adult	miR-375	KLF4	circARRDC3 contributes to interleukin‑13‑induced inflammatory cytokine and mucus production in nasal epithelial cells via the miR‑375/KLF4 axis	Nasal epithelial cells	33313951	Research
Adult	miR-145	RUNX3	In maintaining the balance between Th1 and Th2 responses by targeting the runt-related transcription factor 3 (RUNX3)	Peripheral blood	33152094	Review
Adult	miR-3934	NA	miR-3934 was down-regulated in PBMCs of asthmatic patients and may function as a potential diagnosis biomarker	Peripheral blood mononuclear cells (PBMCs) and serum	33506046	Research
Adult	miR-29c	NA	LncRNA TUG1 facilitates Th2 cell differentiation by targeting the miR-29c/B7-H3 axis on macrophages	Peripheral blood, monocyte	34335559	Research
ACOs	miR-19b-3p, miR-125b-5p, miR-320c	NA	The plasma levels of hsa-miR-19b-3p, hsa-miR-125b-5p and hsa-miR-320c in patients with asthma, COPD and asthma–COPD overlap syndrome (ACOS)	Plasma	34151771	Research
Adult	miR-122-5p	NA	Increased in plasma and sputum supernatant EVs derived from patients with (severe) asthma, and this miRNA correlated with immune cell types in the blood	Plasma	32627209	Research
Adult	miR-19b-3p, miR-320c	NA	NA	Plasma	33349226	Research
Adult	miR-574-5p	NA	Related to incident asthma prediction and vitamin D effect modification	Plasma	33923455	Research
Adult	miR-206	NA	Plasma miR-206, IL-4, IL-13, and INF-γ have potential significance for prognosis of asthma induced pulmonary arterial hypertension	Plasma	33086901	Research
Adult	miR-16, miR-125b, miR-133b, miR-206, miR-299	NA	Plasma miRNAs able to distinguish asthmatics from healthy individuals or those with allergic rhinitis	Plasma	33128813	Review
Adult	miR-122-5p	NA	Levels of miR-122-5p higher in patients with (severe) asthma	Plasma/sputum	34067156	Review
Adult	miR-223, miR-21	NA	Biomarker	Plasma/exosome	33904674	Research
Adult	miR-142-5p and miR-130a-3p	NA	miR-142-5p and miR-130a-3p regulate pulmonary macrophage polarization and asthma airway remodeling	Pulmonary macrophages	32524675	Research
ACOS	miR-15b-5p	NA	Circulating microRNA-15b-5p as a biomarker for asthma–COPD overlap	Serum	32713026	Research
Adult	miR‐28‐3p, miR‐16‐2‐3p, and miR‐210‐3p	NA	Differentially expressed in the serum of severe asthma patients	Serum	34161666	Research
Adult	miR-21, miR-155	NA	Biomarkers for bronchial asthma	Serum	31986951	Research
Adult	miR-1246, miR-5100, miR-338-3p	NFKB2, NFATC3, DUSP1, DUSP2, DUSP5 and DUSP16	Altered expression of miR-1246, miR-5100, and miR-338-3p after 8 weeks of benralizumab administration, which could be used as early response markers	Serum	33525548	Research
Adult	miR-106a, miR-126a, miR-146a, miR-126a, miR-106a, miR-19b	NA	Serum miRNA (miRNA106a and miRNA126a, miRNA146a, 126a, 106a, and 19b) expression correlates with clinical characteristics of asthma and systemic inflammation in an age-dependent manner	Serum	34112152	Research
Adult	miR-125b	NA	Overexpression of miR-125b in severe asthma which was associated with serum IgE and hs-CRP may suggest that this molecule is linked to inflammatory reactions	Serum	34001212	Research
Adult	miR-338-3p	NA	Biomarker	Serum	33808110	Research
Adult	miR-126	NA	Levels of miRNA-126 higher in asthmatics	Serum	34067156	Review
Adult	miR-125b	NA	Levels of miRNA-125b higher in patients and correlating with disease severity	Serum	34067156	Review
Adult	miR-155, -146a, miR-223, -374a	NA	Serum miRNAs correlating to clinical parameters in asthma subgroups	Serum	33128813	Review
Adult	miR-126	DNMT1	Asthma progression	Serum	33488613	Review
Adult	miR-92a	MUC5AC	Up-regulation of miR-92a contributes to blocking goblet cell metaplasia by targeting MUC5AC in asthma	Serum/lung	32571119	Research
Adult	miR-181-5p	NA	Strong inverse correlation between plasma miR-181b-5p and airway eosinophilia	Serum/plasma	33478047	Review
Adult	miR-629-3p, miR-223-3p, miR-142-3p	NA	Associated with severe neutrophilic asthma	Sputum	33214212	Review
Adult	miR-629-3p, miR-223-3p, and miR-142-3p	NA	Significant up-regulation of miR-629-3p, miR-223-3p, and miR-142-3p in sputum of severe asthmatics compared to healthy controls, with the highest levels in patients with neutrophilic asthma	Sputum	32973742	Review

### Circulating and Exosome-Derived miRNAs

Even outside of the airways, miRNAs have showed promise as asthma predictors. Several studies with plasma samples of asthmatic patients have been undertaken to identify dysregulated miRNAs. A study identified miR-19b-3p and miR-320c significantly dysregulated in moderate asthmatic patients in comparison with control group and showed a positive correlation between the expression level of miR-320c and IL-4 levels [49]. Under the influence of vitamin D treatment, a plasma circulating miRNA, miR-574-5p, was discovered to be related with and predictive of asthma [50]. It has been reported that plasma circulating miR-223 and miR-21 had a diagnosis estimation probability of 83 and 76% in moderate asthmatic patients, respectively, and could be employed as biomarkers or for targeted immunotherapies in asthma [51]. It has been shown that plasma miR-206, IL-4, IL-13, and INF-γ has potential significance for prognosis of asthma-induced pulmonary arterial hypertension [52]. It is intriguing to suggest that plasma miR-122-5p can differentiate different subtypes of asthma, such as neutrophilic versus eosinophilic asthma, given its IPA-predicted role in lymphocyte differentiation and function [53]. Plasma miR-206, IL-4, IL-13, and INF-γ have been found to have potential prognostic value in asthma-induced pulmonary arterial hypertension [52].

Recently, miRNAs were utilized to identify asthma subgroups in serum; investigations reported that miR‐28‐3p, miR‐16‐2‐3p, miR‐210‐3p, miR-185, miR-125b, miR-338-3p, and miR-125b were associated with severe asthma [54–56]. Another study found that miR-3934 levels in PBMCs and serum can distinguish asthma patients from controls, particularly severe asthma patients, and that miR-3934 levels in PBMCs were negatively correlated with serum levels of IL-6, IL-8, and IL-33 in asthma patients, respectively [57]. Several biomarker studies have been undertaken to identify extracellular vesicle-derived miRNAs from bronchoalveolar lavage (BAL) as well as cell-specific miRNAs that are dysregulated in asthma. By comparing serum expression levels in asthmatic patients to those in healthy controls and associating their levels with serum IL-4, one study found that miR-21 and miR-155 are promising non-invasive biomarkers in the diagnosis of eosinophilic asthma and its response to therapy [58]. Another study identified miR-1246, miR-5100, and miR-338-3p as biomarkers for predicting the response to the biological drug benralizumab [59]. One study evaluated the effect of aging on serum miRNA expression in asthmatics and found that serum miRNA (miR-146a, miR-126a, miR-106a, and miR-19b) expression correlates with clinical characteristics of asthma and systemic inflammation in an age-dependent manner, implying that miRNA may contribute to asthma pathogenesis differently in elderly and non-elderly patients [60].

Recent in-depth investigations have revealed possible links between miRNA gene targets and asthma pathology, implying that numerous signaling systems could be involved. It is reported that miR-20a-5p targets ATG7-regulated cell death, fibrosis, and inflammation in an ovalbumin (OVA)–induced mouse model of allergic asthma [61]. Another study found that the miR-106b-5p/E2F1/SIX1 signaling pathway could be used to develop asthma therapies [62]. It has been reported that borneol reduces asthma symptoms by inhibiting CD4^+^ T-cell proliferation by down-regulating miR-26a and miR-142-3p [63]. In asthma, up-regulation of miR-92a in the serum leads to the blocking of goblet cell metaplasia by targeting MUC5AC [64]. Still, there is a need to study more miRNA and its target genes for better understanding the asthma pathogenesis.

The exosome plays an important role in chronic asthma. The DDAH1/Wnt/-catenin signaling pathway enhances oxidative stress and inflammatory responses in asthmatic mice via miR-21 secreted by mast cell–derived extracellular vesicles [65]. miR-21-5p in macrophage-derived exosomes targets Smad7 in airway epithelial cells to promote epithelial mesenchymal transition [66]. Exosomes generated from M2 macrophages carry miR-370, which slows asthma progression by inhibiting FGF1 production and the MAPK/STAT1 signaling pathway [67].

Thus, circulating miRNAs have showed potential as non-invasive biomarkers and asthma etiology predictors.

### Asthmatic Airways and Airway Remodeling

Asthma has been associated to airway remodeling, which is a change in the fundamental architecture of the airway walls. These structural changes are characterized by epithelial goblet cell hyperplasia and metaplasia, an increase in bronchial smooth muscles and new blood vessels, and interstitial collagen deposition that extends beyond the thickened lamina reticularis to involve the entire inner airway wall in proportion to disease severity [68]. Several studies were conducted to examine the expression and role of miRNA in airway remodeling. One of the studies showed a role for miR-620 in promoting TGF-β1-induced proliferation of airway smooth muscle cell through controlling PTEN/AKT signaling pathway [69]. The investigators reconstructed circular-RNA-miRNA-mRNA regulatory network using miRNA and mRNA expression data of bronchial brushing samples from asthma patients and healthy patients. Downstream analysis identified the top 10 epithelial RNAs: hsa_circ_0001585, hsa_circ_0078031, hsa_circ_0000552, miR-30a-3p, miR-30d-3p, KIT, CD69, ADRA2A, BPIFA1, and GGH, demonstrating the utility of the epithelial circRNA-miRNA-mRNA network in understanding the pathogenesis of asthma [70]. miR-21 dysregulation in the circulation and airways has been widely observed in allergic asthma and extensively investigated in humans and mice [71, 72]. According to studies, in an ovalbumin-induced allergic asthma mice model, miR-21 inhibition suppresses alveolar M2 macrophages [71], and in human bronchial smooth muscle cells, the miR-21-transforming growth factor 1-Smad7 axis controls the pathogenesis of ovalbumin-induced chronic asthma [72]. According to a study, TUG1 reinforces HMGB1 expression by sequestering miR-181b, which activates the NF-B signaling pathway and promotes airway remodeling in asthmatic mice [73]. An in vitro investigation showed that miR-30b-5p targets phosphatase and tensin homolog deleted on chromosome ten (PTEN) and stimulates the proliferation and migration of human airway smooth muscle cells triggered by platelet-derived growth factor [74]. According to a study, reduced A-to-I editing of miR-200b-3p position 5 in lower airway cells from moderate-to-severe asthmatic individuals may lead to overexpression of SOCS1 and defective cytokine signaling [75]. Interlukin-13-dependent RhoA protein expression is negatively controlled by miR-140-3p in ASMs, according to a study, and the RhoA/Rho-kinase pathway has been suggested as a new target for the therapy of AHR in asthma [76, 77]. miR-149 inhibits TGF-1-induced airway smooth muscle cell proliferation and migration via targeting TRPM7 and altering the downstream MAPK signal pathway [78]. miR-135a reduces asthmatic mice’s airway inflammatory response through modulating the JAK/STAT signaling pathway [79]. Pulmonary macrophage polarization and asthma airway remodeling are regulated by miR-142-5p and miR-130a-3p [80]. By regulating the transforming growth factor-Smad7 pathway, miR-21 inhibition reduces airway inflammation and remodeling [72]. In nicotine-induced airway remodeling, miR-98 suppresses nerve growth factor expression [81].

PRMT1 was found to be a coactivator for STAT1 or RUNX1, which is required for the transcription of pri-let-7i and pri-miR-423 in epithelial cells and could be linked to asthmatic epithelial dysfunction [82]. By targeting miR-143-3p via HMGB1, OIP5AS1 increased Der p1-induced inflammation and apoptosis in BEAS2B cells [83]. TNF receptor-associated factor 6 is targeted by miR-146a-5p, which reduces the inflammatory response and damage of airway epithelial cells [84]. The CD39–extracellular ATP axis, which represents a potentially unique therapeutic target in type 2–high asthma, is targeted by epithelial miR-206, which up-regulates airway IL-25 and TSLP expression [85]. A study discovered that miR-141-3p governs pathological airway mucus production, and in T2-high asthma, miR-141-3p and/or its mRNA targets could be useful therapeutic targets [86]. Airway smooth muscle cell (ASMC) regulation is strongly influenced by epigenetic processes. By modulating miR-149, the lncRNA PVT1 exacerbates asthmatic inflammation and cell-barrier damage [87]. The PVT1-miR-15a-5p/miR-29c-3p-PI3K-Akt-mTOR lncRNA axis has been associated with the development of ozone-induced asthma by stimulating ASMC proliferation and a Th1/Th2 imbalance [88]. Furthermore, another study showed that lncRNA TUG1 facilitates Th2 cell differentiation on macrophages by targeting the miR-29c/B7-H3 axis [89]. The increase of CD38 protein in ASMC of asthmatic patients may be caused by the down-regulation of miR-140-3p produced by IL-13 [76]. Another study found that the miR-375/Krüppel-like factor 4 (KLF4) axis contributes to IL-13-induced inflammatory cytokine and mucus production in nasal epithelial cells (NECs) via circARRDC3 [90] (Table 3).

**Table 3:** List of miRNAs associated with asthmatic airways and airway remodeling

**Type**	**miRNA**	**Target gene**	**Function**	**Sample**	**Reference**	**Review/research**
ASMCs	miR-620	NA	Promotes TGF-β1-induced proliferation of airway smooth muscle cell through controlling PTEN/AKT signaling pathway	ASMCs	32583575	Research
ASMCs	miR-620	NA	miR-620 promotes TGF-β1-induced proliferation of airway smooth muscle cell through controlling PTEN/AKT signaling pathway	ASMCs	32583575	Research
ASMCs	miR-145	MMP-2, MMP-9	Binds KLF5 3ʹUTR	ASMCs	34359876	Review
ASMCs	miR-143-3p	NFATc1	Promotes collagen 1 and fibronectin expressions, leading to elevated ASM cell proliferation and up-regulation of CDK4 and cyclin D1 expressions	ASMCs	34359876	Review
ASMCs	miR-378	NA	miR-378 is elevated in ASM cells from asthmatic patients and, via MAPK and calcium signaling, can up-regulate collagen I and fibronectin expression	ASMCs	34359876	Review
ASMCs	miR-204-5p	Six1	miR-204-5p has also been shown to be down-regulated in ASM cells from asthmatic patients and promotes the expressions of fibronectin and collagen III via the Six1 gene (a TGF-β1 inducible gene)	ASMCs	34359876	Review
ASMCs	miR-145	KLF4	miR-145 was significantly elevated and led to increased collagen I and myosin heavy chain expression through negative regulation of the transcription factor Krüppel-like factor 4 (KLF4) protein and downstream activation of MMP-2 and MMP-9	ASMCs	34359876	Review
ASMCs	miR-25	Collagen XI	Inhibition of miR-25 in IL-1β, TNF-α and IFN-γ-stimulated ASM cells, had a greater than twofold down regulatory effect on collagen XI expression, and to a lesser extent the expressions of collagen (V and XV), fibronectin, MMP-9, and integrin (αm and β2), by stimulating KLF4 expression	ASMCs	34359876	Review
ASMCs	miR-181a	Collagen I and fibronectin	miR-181a expression in ASM, leading to the overexpression of collagen I and fibronectin, via the Akt signaling pathway	ASMCs	34359876	Review
ASMCs	miR-142	NA	Overexpressed in ASM cells derived from an asthma rat model and inhibits TGF-β expression via epidermal growth factor receptor (EGFR) signaling	ASMCs	34359876	Review
ASMCs	miR-146a, miR-221	NA	Regulating airway smooth muscle (ASM) cell function	ASMCs	34134446	Review
ASMCs	miR-140-3p, miR-708, miR-142-3p	NA	ASM cell hyperplasia and hypertrophy; Th2 responses and IgE production	ASMCs	34134446	Review
ASMCs	miR-10a	NA	Regulating the proliferation of ASM cells via the PI3K pathway	ASMCs	33152094	Review
ASMCs	miR-140-3p	CD38, CCL11, CXCL12, CXCL10, CCL5, CXCL8	CD38 expression, chemokine regulation, inflammation, and ASMC proliferation in asthma	ASMCs	33488613	Review
ASMCs	miR-145	KLF4	ASMC proliferation and migration	ASMCs	33488613	Review
ASMCs	miR-146a-5p	UBD, CXCL10, CXCL8, CCL20, UCA1	Mucus production	ASMCs	33488613	Review
ASMCs	miR-638	NR4A3, CCND1	ASMC proliferation and migration	ASMCs	33488613	Review
ASMCs	miR-708	CD38, CCL11, CXCL10, CCL2, CXCL8, JNK, MAPK, PTEN/AKT signaling pathways	CD38 expression, chemokine regulation, inflammation and ASMC proliferation in asthma	ASMCs	33488613	Review
ASMCs	miR-146a/b	PTGS2, IL1B, NOTCH5	Regulation of inflammation, macrophage M2 Polarization	ASMCs	33488613	Review
ASMCs	miR-19	Collagen I, fibronectin	miR-19 is decreased in ASM cells from asthmatic patients and induces elevated expression of collagen I, fibronectin and arginine methyltransferase activity through the ERK1/MAPK signaling pathway	ASMCs	34359876	Review
Benralizumab	miR-21-5p	HDAC2, NFE2L2, GLCCI1, PTEN, NR3C1	Benralizumab restores gene and microRNA expression involved in steroid sensitivity in severe asthma	NA	33738833	Research
ASMCs	miR-30b-5p	PTEN	miR-30b-5p activates the PI3K/AKT pathway by targeting PTEN to facilitate PDGF-induced dysfunction of ASM cells	ASMCs	34251961	Research
Bronchial epithelial brushing (HBECs) tissue	miR-19a	TGFBR2	miR-19a to enhance proliferation of BECs in severe asthma through targeting TGF-β receptor 2 gene (*TGFBR2*) mRNA	Bronchial epithelial brushing (HBECs) tissue	32973742	Review
Mice	miR-590-5p	FGF1	TUG1 via sponging miR590-5p/FGF1 promoted airway smooth muscle cells proliferation and migration in asthma	NA	32777705	Review
Mice	miR-21	IL-12, STAT4	Axis of miR-22/IL-12/STAT4 participates in development of allergic asthma	NA	32777705	Review
Mice	miR-21	IL-12p35	miR-21 through the IL-13Rα1-independent pathway overexpressed in mouse allergic asthma	NA	32777705	Review
Mice	miR-20b	NA	Intranasal administration of miR-20b increased the percentage of Gr1 + CD11b + myeloid-derived suppressor cells (MDSCs) and increased TGF-β in the lung of asthmatic mice	NA	32777705	Review
Mice	miR-20b	NA	miRNA-20b promotes accumulation of CD11b + Ly6G + Ly6Clow MDSCs in asthmatic mice	NA	32777705	Review
Mice	miR-485	Smurf2	miR-485 targeting Smurf2 through the TGF-β/Smads signaling pathway, suppresses cell proliferation and promotes cell apoptosis in mice with chronic asthma	NA	32777705	Review
Mice	miR-410	IL-4, IL-13	Intranasal miR‑410 targeting IL-4/IL-13 attenuates airway inflammation in OVA‑induced asthmatic mice	NA	32777705	Review
Mice	let-7	IL13	Regulation of asthmatic hyper-response	Lung	33488613	Review
Mice	miR-487b	NA	miR-487b in activating and regulating macrophages in innate immune responses including pro-inflammatory effects through the induction of IL-33 transcripts	NA	33152094	Review
Murine model	miR-155	IL-33	miR-155 required for allergen-induced ILC2 expansion and IL-33 production, asthma mouse model	NA	32777705	Review
Murine model	miR-21	HDAC2	miR-21 induced in the lung by infection, during steroid-insensitive allergic airway disease in BALB/c. miR-21. Amplifies PI3K–mediated suppression of HDAC2 driving severe steroid-insensitive experimental asthma	NA	32777705	Review
NA	miR-146a	IL-5, IL-13	miR-146a decreases influx of inflammatory cells into lung, suppresses OVA-specific IgE and Th2 cytokines, attenuating airway hyper-responsiveness and allergic inflammation	NA	32777705	Review
NA	miR-21	PTEN	miR-21 through PTEN/PI3K/Akt signaling pathway modulates human ASMC proliferation and migration in asthma	NA	32777705	Review
NA	miR-155	COX-2	miR-155 assists overexpression of COX-2 in asthmatic ASMCs	NA	32777705	Review
NA	miR-155	chemokine expression (CCL5, CCL11, CCL26, CXCL8, and CXCL10)	miR-155 as a novel target in allergic asthma	Bronchial epithelial brushing (HBECs) tissue	33152094	Review
NA	miR-3162-3p	CTNNB1	NA	Mice	33488613	Review
NA	miR-155	IL-6, KRAS, IL-17, IL-21, IL-6,	Anti-inflammatory	NA	33378051	Review
NA	miR-21a	IL-1beta	NA	NA	33378051	Review
NA	miR-146a	IL-17, IL-21, IL-6	NA	NA	33378051	Review

**Fig. 2:**
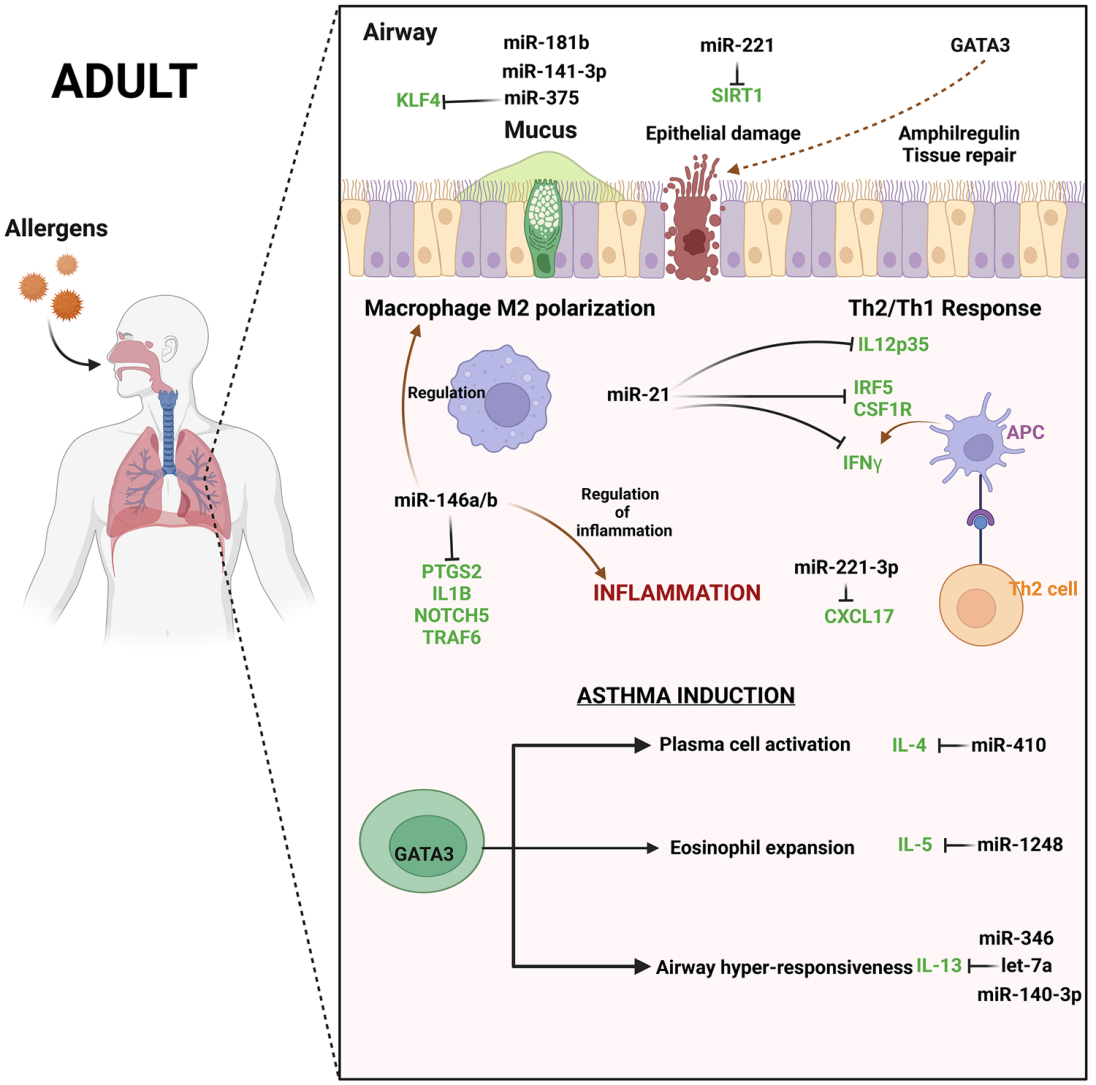
Illustrating miRNAs and their target genes associated with adult asthma

Together, emerging data indicate that the miRNAs play a crucial role in asthmatic airways and airway remodeling, performing an integral role in post-transcriptional regulation within the complex biological network (Tables 2 and 3; Fig. 2).

### Asthma–COPD Overlap Syndrome

Recently, several studies were conducted to identify miRNAs as biomarkers for distinguishing patients with ACOS (asthma–COPD overlap syndrome) from patients with COPD or asthma. Hirai et al. proposed miR-15b-5p as a potential marker for identifying patients with ACOS. When miR-15b-5p, serum periostin, and YKL-40 were combined, it can improve diagnosis accuracy for ACOS (AUROC, 0.80) [91]. Another study depicted free-circulating miR-19b-3p, miR-125b-5p, and miR-320c in the blood plasma as three potential biomarkers for the diagnosis of COPD, bronchial asthma, and ACOS [92]. The collected literature reflects potential use of miRNAs as a tool for distinguishing these three very similar diseases: COPD, asthma, and ACOS.

### miRNA and Virus-Induced Exacerbations in Asthma

**Fig. 3:**
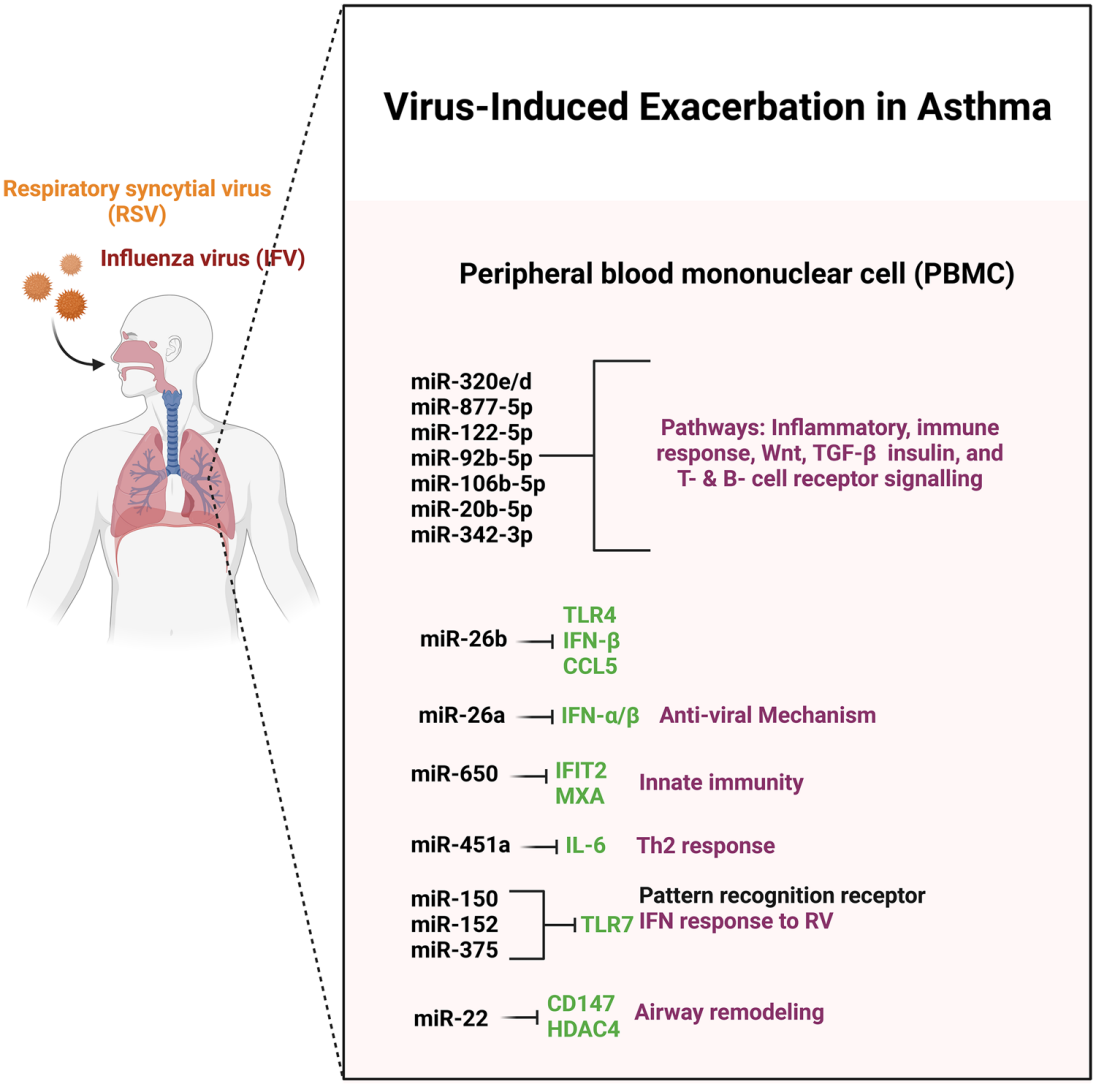
miRNAs associated with virus-induced exacerbation in asthma

Human respiratory virus (RV), human respiratory syncytial virus (RSV), and influenza viruses are all common viruses that attack the respiratory system. These viruses are known to induce illness and exacerbations in asthmatics [93]. The study found that suppressing STIM1 alleviated influenza A virus (IAV)–induced lung epithelial cell inflammation by inactivating NLRP3 (NLR Family Pyrin Domain Containing 3) and the inflammasome and increasing miR-223 expression. These findings may aid researchers to better understand the mechanism of influenza A virus (IAV)–induced lung injury and aid in IAV infection treatment [94]. The induction of MUC5AC synthesis by reduced miR-34b/c-5p was partly mediated by activation of c-Jun in RSV-infected HBECs. These findings shed light on the mechanism of mucus obstruction following RSV infection and point to potential therapeutic targets for RSV infection and airway obstruction [95]. In vivo, miR-122 enhances RV-induced asthma by suppressing its target SOCS1 [96]. In addition, influenza virus induces miR-146a. By directly targeting the tumor necrosis factor receptor association factor 6 (TRAF6), infection and down-regulation of miR-146a have been demonstrated to decrease influenza A virus multiplication by increasing IFN type 1 responses [97]. These findings point to miRNA modulation of immune responses to respiratory viruses (Fig. 3), and it is tempting to believe that miRNAs that alter virus replication play a key role in asthma exacerbations caused by viruses (Table 4).

**Table 4:** Details of miRNAs associated with virus-induced asthma

**miRNA ID**	**Target gene**	**Function**	**Sample**	**Reference (PubMed ID)**	**Review/research**
miR-146a/b	CCL5, IL-8 and CXCL1, IFNL1	Strong anti-inflammatory effect on RV infection and allergic airway inflammation	Human bronchial epithelial cells (HBECs)/mouse	34185416	Research
miR-29, -29c, -136, 449b, and let-7c	NA	Increased expression in influenza A virus’s infection	A549 cells	33255348	Review
miR-155	NA	Inhibition of miR-155 in human bronchial epithelial cells resulted in an increased viral replication of RV-1B	Human bronchial epithelial cells (HBECs)/mouse	33255348	Review
miR-18a, -27a, -128 and -155	IL-6 and CXCL8	NA	Human bronchial epithelial cells (HBECs)/mouse	33255348	Review
miR-24, -124a, and -744	NA	Antiviral effects on influenza A virus in the human lung epithelial cell line A549	Human lung epithelial cell line A549	33255348	Review
miR-124a and -744	NA	Antiviral effects in RSV infection	Human lung epithelial cell line A549	33255348	Review
miR-146a	TRAF6	Down-regulation of miR-146a was shown to inhibit influenza A virus replication by enhancing IFN type 1 responses by directly targeting the tumor necrosis factor receptor association factor 6	NA	33255348	Review
miR-223	NA	STIM1 mediates IAV-induced inflammation of lung epithelial cells by regulating NLRP3 and inflammasome activation via targeting miR-223	Human bronchial epithelial cells (HBECs)/mouse	33278394	Research
miR-34b/c-5p	NA	Respiratory syncytial virus infection-induced mucus secretion by down-regulation of miR-34b/c-5p expression in airway epithelial cells	Human bronchial epithelial cells (HBECs)/mouse	32939938	Research
miR-146	TRAF6	Dual role of the miR-146 family in rhinovirus-induced airway inflammation and allergic asthma exacerbation	Human bronchial epithelial cells (HBECs)/mouse	34185416	Research
miR-122	SOCS1	Promotes virus-induced lung disease by targeting SOCS1	Lung tissue	33830082	Research

## Conclusion

It is difficult to accurately compare childhood and adult-onset asthma due to existing gaps in the literature and we acknowledge this limitation. In addition, because some findings are reported more in adults, this does not necessarily mean they are more prevalent, but rather a possible manifestation of publication bias. In this review, we tried to group the miRNAs from recent publications broadly into adult and childhood asthma and further sub-categorized into exosome derived, plasma/serum, ACOs, and role of miRNA in virus-induced exacerbations in asthma.

## References

[1] Tarlo SM, Balmes J, Balkissoon R, Beach J, Beckett W, Bernstein D (2008). Diagnosis and management of work-related asthma: American College of Chest Physicians Consensus Statement. Chest.

[2] GAN. The Global Asthma Report. Vol. 9, Policy Studies. Auckland, New Zealand; 2018.

[3] Bateman ED, Hurd SS, Barnes PJ, Bousquet J, Drazen JM, FitzGerald JM (2008). Global strategy for asthma management and prevention: GINA executive summary. Eur Respir J.

[4] Eder W, Ege MJ, von Mutius E (2006). The asthma epidemic. N Engl J Med.

[5] Ivanova O, Richards LB, Vijverberg SJ, Neerincx AH, Sinha A, Sterk PJ, et al. What did we learn from multiple omics studies in asthma? Allergy [Internet]. 2019;74(11):2129–45. Available from: 10.1111/all.13833. 10.1111/all.13833 31004501

[6] Gautam Y, Johansson E, Mersha TB. Multi-Omics Profiling Approach to Asthma: An Evolving Paradigm. J Pers Med. 2022 Jan 7;12(1):66. 10.3390/jpm12010066. 10.3390/jpm12010066 PMC8778153 35055381

[7] Kim VN, Han J, Siomi MC (2009). Biogenesis of small RNAs in animals. Nat Rev Mol Cell Biol.

[8] Hoefel G, Tay H, Foster P. MicroRNAs in lung diseases. Chest. 2019 Nov;156(5):991–1000. 10.1016/j.chest.2019.06.008. Epub 2019 Jun 27. 10.1016/j.chest.2019.06.008 31255581

[9] Ardekani AM, Naeini MM. The role of MicroRNAs in human diseases. Avicenna J Med Biotechnol. 2010;2(4):161–79. PMC3558168 23407304

[10] Cañas JA, Rodrigo-Muñoz JM, Gil-Martínez M, Sastre B, del Pozo V (2021). Exosomes: a key piece in asthmatic inflammation. Int J Mol Sci.

[11] Bélanger É, Madore A-M, Boucher-Lafleur A-M, Simon M-M, Kwan T, Pastinen T (2020). Eosinophil microRNAs play a regulatory role in allergic diseases included in the atopic march. Int J Mol Sci.

[12] Tan BWQ, Sim WL, Cheong JK, Kuan WS, Tran T, Lim HF (2020). MicroRNAs in chronic airway diseases: clinical correlation and translational applications. Pharmacol Res.

[13] Usman K, Hsieh A, Hackett T-L (2021). The role of miRNAs in extracellular matrix repair and chronic fibrotic lung diseases. Cells.

[14] Adcock IM, Mumby S (2021). MicroRNAs in human disease: commentary. Iran J Allergy Asthma Immunol.

[15] Alashkar Alhamwe B, Potaczek DP, Miethe S, Alhamdan F, Hintz L, Magomedov A (2021). Extracellular vesicles and asthma-more than just a co-existence. Int J Mol Sci.

[16] Cañas JA, Rodrigo-Muñoz JM, Sastre B, Gil-Martinez M, Redondo N, Del Pozo V (2020). MicroRNAs as potential regulators of immune response networks in asthma and chronic obstructive pulmonary disease. Front Immunol.

[17] Paul S, Ruiz-Manriquez LM, Ledesma-Pacheco SJ, Benavides-Aguilar JA, Torres-Copado A, Morales-Rodríguez JI (2021). Roles of microRNAs in chronic pediatric diseases and their use as potential biomarkers: a review. Arch Biochem Biophys.

[18] Akbari Dilmaghnai N, Shoorei H, Sharifi G, Mohaqiq M, Majidpoor J, Dinger ME (2021). Non-coding RNAs modulate function of extracellular matrix proteins. Biomed Pharmacother.

[19] Calvén J, Ax E, Rådinger M (2020). The airway epithelium–a central player in asthma pathogenesis. Int J Mol Sci.

[20] Benincasa G, DeMeo DL, Glass K, Silverman EK, Napoli C (2021). Epigenetics and pulmonary diseases in the horizon of precision medicine: a review. Eur Respir J.

[21] Shastri MD, Chong WC, Dua K, Peterson GM, Patel RP, Mahmood MQ (2021). Emerging concepts and directed therapeutics for the management of asthma: regulating the regulators. Inflammopharmacology.

[22] •• Weidner J, Bartel S, Kılıç A, Zissler UM, Renz H, Schwarze J, et al. Spotlight on microRNAs in allergy and asthma. Allergy. 2021;76(6):1661–78. **COMMENT: A good collection of miRNAs associated with Asthma.** 10.1111/all.14646 PMC8246745 33128813

[23] Alashkar Alhamwe B, Miethe S, von Strandmann E, Potaczek DP, Garn H (2020). Epigenetic regulation of airway epithelium immune functions in asthma. Front Immunol.

[24] Ghafouri-Fard S, Shoorei H, Taheri M, Sanak M (2020). Emerging role of non-coding RNAs in allergic disorders. Biomed Pharmacother.

[25] Casciaro M, Di Salvo E, Pioggia G, Gangemi S (2020). Microbiota and microRNAs in lung diseases: mutual influence and role insights. Eur Rev Med Pharmacol Sci.

[26] Kuruvilla ME, Lee FE-H, Lee GB (2019). Understanding asthma phenotypes, endotypes, and mechanisms of disease. Clin Rev Allergy Immunol.

[27] Trivedi M, Denton E. Asthma in children and adults—what are the differences and what can they tell us about asthma? Front Pediatr. 2019 Jun 25;7:256. 10.3389/fped.2019.00256. 10.3389/fped.2019.00256 PMC6603154 31294006

[28] Sood A, Qualls C, Schuyler M, Arynchyn A, Alvarado JH, Smith LJ, et al. Adult-onset asthma becomes the dominant phenotype among women by age 40 years. The longitudinal CARDIA study. Ann Am Thorac Soc. 2013 Jun;10(3):188–97. 10.1513/AnnalsATS.201212-115OC. 10.1513/AnnalsATS.201212-115OC PMC3960903 23802814

[29] O’Brien J, Hayder H, Zayed Y, Peng C. Overview of MicroRNA biogenesis, mechanisms of actions, and circulation. Front Endocrinol (Lausanne) [Internet]. 2018;9. Available from: 10.3389/fendo.2018.00402. 10.3389/fendo.2018.00402 PMC6085463 30123182

[30] Liu X, Chen X, Yu X, Tao Y, Bode AM, Dong Z, et al. Regulation of microRNAs by epigenetics and their interplay involved in cancer. J Exp Clin Cancer Res [Internet]. 2013;32(1):96. Available from: 10.1186/1756-9966-32-96. 10.1186/1756-9966-32-96 PMC3874662 24261995

[31] Rodrigo-Muñoz JM, Cañas JA, Sastre B, Rego N, Greif G, Rial M (2019). Asthma diagnosis using integrated analysis of eosinophil microRNAs. Allergy.

[32] Taka S, Tzani-Tzanopoulou P, Wanstall H, Papadopoulos NG (2020). MicroRNAs in asthma and respiratory infections: identifying common pathways. Allergy Asthma Immunol Res.

[33] Wang J, Chen J, Sen S (2016). MicroRNA as biomarkers and diagnostics. J Cell Physiol.

[34] Mori MA, Ludwig RG, Garcia-Martin R, Brandão BB, Kahn CR. Extracellular miRNAs: from biomarkers to mediators of physiology and disease. Cell Metab [Internet]. 2019/08/22. 2019;30(4):656–73. Available from: https://pubmed.ncbi.nlm.nih.gov/31447320. 10.1016/j.cmet.2019.07.011 PMC6774861 31447320

[35] Simpson CR, Sheikh A. Trends in the epidemiology of asthma in England: a national study of 333,294 patients. J R Soc Med. 2010 Mar;103(3):98–106. 10.1258/jrsm.2009.090348. 10.1258/jrsm.2009.090348 PMC3072257 20200181

[36] Ibrahim AA, Ramadan A, Wahby AA, Draz IH, El Baroudy NR, Abdel Hamid TA (2020). Evaluation of miR-196a2 expression and Annexin A1 level in children with bronchial asthmaEvaluation of miR-196a2 expression and Annexin A1 level in children. Allergol Immunopathol (Madr).

[37] Wang T, Zhou Q, Shang Y (2021). Downregulation of miRNA-451a promotes the differentiation of CD4+ T cells towards Th2 cells by upregulating ETS1 in childhood asthma. J Innate Immun.

[38] Wang AL, Li J, Kho AT, McGeachie MJ, Tantisira KG (2021). Enhancing the prediction of childhood asthma remission: integrating clinical factors with microRNAs. J Allergy Clin Immunol.

[39] Tiwari A, Li J, Kho AT, Sun M, Lu Q, Weiss ST (2021). COPD-associated miR-145-5p is downregulated in early-decline FEV1 trajectories in childhood asthma. J Allergy Clin Immunol.

[40] Elnady HG, Sherif LS, Kholoussi NM, Ali Azzam M, Foda AR, Helwa I (2020). Aberrant expression of immune-related MicroRNAs in pediatric patients with asthma. Int J Mol Cell Med.

[41] Li W, Wang X, Sun S, An H (2021). Long non-coding RNA colorectal neoplasia differentially expressed correlates negatively with miR-33a and miR-495 and positively with inflammatory cytokines in asthmatic children. Clin Respir J.

[42] Yin H, Liu MH, Gao F, Shang HM (2021). Pro-inflammatory and pro-fibrotic role of long non-coding RNA RMRP in pediatric asthma through targeting microRNA-206/CCL2 axis. J Biol Regul Homeost Agents.

[43] Tiwari A, Wang AL, Li J, Lutz SM, Kho AT, Weiss ST (2021). Seasonal variation in miR-328-3p and let-7d-3p are associated with seasonal allergies and asthma symptoms in children. Allergy Asthma Immunol Res.

[44] Sharma S, Kho AT, Chhabra D, Haley K, Vyhlidal C, Gaedigk R (2020). Effect of intrauterine smoke exposure on microRNA-15a expression in human lung development and subsequent asthma risk. Healthc.

[45] Zhai C, Wang D. Baicalin regulates the development of pediatric asthma via upregulating microRNA-103 and mediating the TLR4/NF-κB pathway. J Recept Signal Transduct Res. 2021;1–11. 10.1080/10799893.2021.1900865 33730981

[46] Dong J, Sun D, Lu F (2021). Association of two polymorphisms of miRNA-146a rs2910164 (G > C) and miRNA-499 rs3746444 (T > C) with asthma: a meta-analysis. J Asthma.

[47] Rahbarghazi R, Keyhanmanesh R, Rezaie J, Mirershadi F, Heiran H, Saghaei Bagheri H (2021). c-kit+ cells offer hopes in ameliorating asthmatic pathologies via regulation of miRNA-133 and miRNA-126. Iran J Basic Med Sci.

[48] Wang K, Zhu H, Yang L, Xu Q, Ren F (2021). miR-139 promotes homing of bone marrow mesenchymal stem cells (BMSCs) to lung tissues of asthmatic rats to inhibit inflammatory response of Th2 cells by down-regulating Notch1/Hes1 pathway. Xi Bao Yu Fen Zi Mian Yi Xue Za Zhi.

[49] Aripova A, Akparova A, Bersimbaev R (2020). Moderate bronchial asthma. MicroRNA.

[50] Li J, Tiwari A, Mirzakhani H, Wang AL, Kho AT, McGeachie MJ (2021). Circulating MicroRNA: incident asthma prediction and vitamin D effect modification. J Pers Med.

[51] Rostami Hir S, Alizadeh Z, Mazinani M, Mahlooji Rad M, Fazlollahi MR, Kazemnejad A (2021). Exosomal MicroRNAs as biomarkers in allergic asthma. Iran J Allergy Asthma Immunol.

[52] Li S, Ma X, Xie J, Yan X, Sun W (2021). MicroRNA-206, IL-4, IL-13, and INF-γ levels in lung tissue and plasma are increased by the stimulation of particulate matter with a diameter of ≤2.5μm, and are associated with the poor prognosis of asthma induced pulmonary arterial hypert. Clin Exp Hypertens.

[53] Bahmer T, Krauss-Etschmann S, Buschmann D, Behrends J, Watz H, Kirsten A-M (2021). RNA-seq-based profiling of extracellular vesicles in plasma reveals a potential role of miR-122-5p in asthma. Allergy.

[54] Kyyaly MA, Sanchez-Elsner T, He P, Sones CL, Arshad SH, Kurukulaaratchy RJ (2021). Circulating miRNAs-A potential tool to identify severe asthma risk?. Clin Transl Allergy.

[55] Atashbasteh M, Mortaz E, Mahdaviani SA, Jamaati H, Allameh A (2021). Expression levels of plasma exosomal miR-124, miR-125b, miR-133b, miR-130a and miR-125b-1-3p in severe asthma patients and normal individuals with emphasis on inflammatory factors. Allergy Asthma Clin Immunol.

[56] Rial MJ, Cañas JA, Rodrigo-Muñoz JM, Valverde-Monge M, Sastre B, Sastre J (2021). Changes in serum MicroRNAs after anti-IL-5 biological treatment of severe asthma. Int J Mol Sci.

[57] Wang W, Wang J, Chen H, Zhang X, Han K (2021). Downregulation of miR-3934 in peripheral blood mononuclear cells of asthmatic patients and its potential diagnostic value. Biomed Res Int.

[58] ElKashef SMMAE, Ahmad SEA, Soliman YMA, Mostafa MS (2021). Role of microRNA-21 and microRNA-155 as biomarkers for bronchial asthma. Innate Immun.

[59] Cañas JA, Valverde-Monge M, Rodrigo-Muñoz JM, Sastre B, Gil-Martínez M, García-Latorre R (2021). Serum microRNAs as tool to predict early response to benralizumab in severe eosinophilic asthma. J Pers Med..

[60] Wardzyńska A, Pawełczyk M, Rywaniak J, Makowska J, Jamroz-Brzeska J, Kowalski ML (2021). Circulating miRNA expression in asthmatics is age-related and associated with clinical asthma parameters, respiratory function and systemic inflammation. Respir Res.

[61] Yu Y, Men S, Zhang Y (2021). miR-20a-5p ameliorates ovalbumin (OVA)-induced mouse model of allergic asthma through targeting ATG7-regulated cell death, fibrosis and inflammation. Int Immunopharmacol.

[62] Liu S, Chen X, Zhang S, Wang X, Du X, Chen J (2021). miR-106b-5p targeting SIX1 inhibits TGF-β1-induced pulmonary fibrosis and epithelial-mesenchymal transition in asthma through regulation of E2F1. Int J Mol Med.

[63] Wang J-Y, Dong X, Yu Z, Ge L, Lu L, Ding L (2021). Borneol inhibits CD4 + T cells proliferation by down-regulating miR-26a and miR-142-3p to attenuate asthma. Int Immunopharmacol.

[64] Dai J, Ma B, Wen X, Yang Z, Yue Y (2020). Upregulation of miR-92a contributes to blocking goblet cell metaplasia by targeting MUC5AC in asthma. J Recept Signal Transduct Res.

[65] Zou Y, Zhou Q, Zhang Y (2021). MicroRNA-21 released from mast cells-derived extracellular vesicles drives asthma in mice by potentiating airway inflammation and oxidative stress. Am J Transl Res.

[66] Li X, Yang N, Cheng Q, Zhang H, Liu F, Shang Y (2021). MiR-21-5p in macrophage-derived exosomes targets Smad7 to promote epithelial mesenchymal transition of airway epithelial cells. J Asthma Allergy.

[67] Li C, Deng C, Zhou T, Hu J, Dai B, Yi F (2021). MicroRNA-370 carried by M2 macrophage-derived exosomes alleviates asthma progression through inhibiting the FGF1/MAPK/STAT1 axis. Int J Biol Sci.

[68] Hough KP, Curtiss ML, Blain TJ, Liu R-M, Trevor J, Deshane JS, et al. Airway remodeling in asthma. Front Med [Internet]. 2020;7. Available from: 10.3389/fmed.2020.00191. 10.3389/fmed.2020.00191 PMC7253669 32509793

[69] Chen H, Guo S-X, Zhang S, Li X-D, Wang H, Li X-W (2020). MiRNA-620 promotes TGF-β1-induced proliferation of airway smooth muscle cell through controlling PTEN/AKT signaling pathway. Kaohsiung J Med Sci.

[70] Chen D, Wu W, Yi L, Feng Y, Chang C, Chen S (2021). A potential circRNA-miRNA-mRNA regulatory network in asthmatic airway epithelial cells identified by integrated analysis of microarray datasets. Front Mol Biosci.

[71] Lee HY, Hur J, Kang JY, Rhee CK, Lee SY (2021). MicroRNA-21 inhibition suppresses alveolar M2 macrophages in an ovalbumin-induced allergic asthma mice model. Allergy Asthma Immunol Res.

[72] Hur J, Rhee CK, Lee SY, Kim YK, Kang JY (2021). MicroRNA-21 inhibition attenuates airway inflammation and remodelling by modulating the transforming growth factor β-Smad7 pathway. Korean J Intern Med.

[73] Huang W, Yu C, Liang S, Wu H, Zhou Z, Liu A (2021). Long non-coding RNA TUG1 promotes airway remodeling and mucus production in asthmatic mice through the microRNA-181b/HMGB1 axis. Int Immunopharmacol.

[74] Wang W, Guo J, Wang Y (2021). MicroRNA-30b-5p promotes the proliferation and migration of human airway smooth muscle cells induced by platelet-derived growth factor by targeting phosphatase and tensin homolog deleted on chromosome ten. Bioengineered.

[75] Magnaye KM, Naughton KA, Huffman J, Hogarth DK, Naureckas ET, White SR (2021). A-to-I editing of miR-200b-3p in airway cells is associated with moderate-to-severe asthma. Eur Respir J.

[76] Chiba Y, Ando Y, Kato Y, Hanazaki M, Sakai H. Down-regulation of miR-140–3p is a cause of the interlukin-13-induced up-regulation of RhoA protein in bronchial smooth muscle cells. Small GTPases. 2021;1–6. 10.1080/21541248.2021.1872318 PMC9707530 33427568

[77] Chiba Y, Ando Y, Fujii S, Miyakawa Y, Suto W, Kamei J (2021). Downregulation of miR-140-3p is a cause of upregulation of RhoA protein in bronchial smooth muscle of murine experimental asthma. Am J Respir Cell Mol Biol.

[78] Zhu Z, Zhang L, Jiang T, Qian Y, Sun Y, Zhang Q (2020). MiR-149 attenuates the proliferation and migration of TGF-β1-induced airway smooth muscle cells by targeting TRPM7 and affecting downstream MAPK signal pathway. Acta Biochim Pol.

[79] Huang X-P, Qin C-Y, Gao Y-M (2021). miR-135a inhibits airway inflammatory response in asthmatic mice via regulating JAK/STAT signaling pathway. Braz J Med Biol Res.

[80] Shi J, Chen M, Ouyang L, Wang Q, Guo Y, Huang L (2020). miR-142-5p and miR-130a-3p regulate pulmonary macrophage polarization and asthma airway remodeling. Immunol Cell Biol.

[81] Wongtrakool C, Ko J, Jang AJ, Grooms K, Chang S, Sylber C (2020). MicroRNA-98 reduces nerve growth factor expression in nicotine-induced airway remodeling. J Biol Chem.

[82] Zhai W, Sun H, Li Z, Li L, Jin A, Li Y (2021). PRMT1 modulates processing of asthma-related primary MicroRNAs (pri-miRNAs) into mature miRNAs in lung epithelial cells. J Immunol.

[83] Cai X-J, Huang L-H, Zhu Y-K, Huang Y-J (2020). LncRNA OIP5-AS1 aggravates house dust mite-induced inflammatory responses in human bronchial epithelial cells via the miR-143-3p/HMGB1 axis. Mol Med Rep.

[84] Yan F, Wufuer D, Ding J, Wang J (2021). MicroRNA miR-146a-5p inhibits the inflammatory response and injury of airway epithelial cells via targeting TNF receptor-associated factor 6. Bioengineered.

[85] Zhang K, Feng Y, Liang Y, Wu W, Chang C, Chen D, et al. Epithelial miR-206 targets CD39/extracellular ATP to upregulate airway IL-25 and TSLP in type 2-high asthma. JCI Insight. 2021;6(11). 10.1172/jci.insight.148103 PMC8262281 33945508

[86] Siddiqui S, Johansson K, Joo A, Bonser LR, Koh KD, Le Tonqueze O, et al. Epithelial miR-141 regulates IL-13-induced airway mucus production. JCI Insight. 2021;6(5). 10.1172/jci.insight.139019 PMC8021117 33682796

[87] Ma L, Zhang Q, Hao J, Wang J, Wang C (2020). LncRNA PVT1 exacerbates the inflammation and cell-barrier injury during asthma by regulating miR-149. J Biochem Mol Toxicol.

[88] Wei Y, Han B, Dai W, Guo S, Zhang C, Zhao L (2020). Exposure to ozone impacted Th1/Th2 imbalance of CD4+ T cells and apoptosis of ASMCs underlying asthmatic progression by activating lncRNA PVT1-miR-15a-5p/miR-29c-3p signaling. Aging (Albany NY).

[89] Sun H, Wang T, Zhang W, Dong H, Gu W, Huang L (2021). LncRNATUG1 facilitates Th2 cell differentiation by targeting the miR-29c/B7-H3 axis on macrophages. Front Immunol.

[90] Wang T, Wang P, Chen D, Xu Z, Yang L (2021). circARRDC3 contributes to interleukin-13-induced inflammatory cytokine and mucus production in nasal epithelial cells via the miR-375/KLF4 axis. Mol Med Rep.

[91] Hirai K, Shirai T, Shimoshikiryo T, Ueda M, Gon Y, Maruoka S (2021). Circulating microRNA-15b-5p as a biomarker for asthma-COPD overlap. Allergy.

[92] Bersimbaev R, Aripova A, Bulgakova O, Kussainova A, Akparova A, Izzotti A (2021). The plasma levels of hsa-miR-19b-3p, hsa-miR-125b-5p, and hsamiR- 320c in patients with asthma, COPD and Asthma-COPD Overlap Syndrome (ACOS). MicroRNA.

[93] Jartti T, Bønnelykke K, Elenius V, Feleszko W (2020). Role of viruses in asthma. Semin Immunopathol.

[94] Liu C-C, Miao Y, Chen R-L, Zhang Y-Q, Wu H, Yang S-M (2021). STIM1 mediates IAV-induced inflammation of lung epithelial cells by regulating NLRP3 and inflammasome activation via targeting miR-223. Life Sci.

[95] Du X, Yang Y, Xiao G, Yang M, Yuan L, Qin L (2020). Respiratory syncytial virus infection-induced mucus secretion by down-regulation of miR-34b/c-5p expression in airway epithelial cells. J Cell Mol Med.

[96] Collison AM, Sokulsky LA, Kepreotes E, de Siqueira A, Morten M, Edwards MR, et al. miR-122 promotes virus-induced lung disease by targeting SOCS1. JCI Insight. 2021;6(7). 10.1172/jci.insight.127933 PMC8119205 33830082

[97] Laanesoo A, Urgard E, Periyasamy K, Laan M, Bochkov YA, Aab A (2021). Dual role of the miR-146 family in rhinovirus-induced airway inflammation and allergic asthma exacerbation. Clin Transl Med.

